# Establishment of baseline haematology and biochemistry parameters in wild adult African penguins (*Spheniscus demersus*)

**DOI:** 10.4102/jsava.v86i1.1198

**Published:** 2015-03-25

**Authors:** Nola J. Parsons, Adam M. Schaefer, Stephen D. van der Spuy, Tertius A. Gous

**Affiliations:** 1 Southern African Foundation for the Conservation of Coastal Birds (SANCCOB), South Africa; 2 Harbor Branch Oceanographic Institution, Florida Atlantic University, United States; 3 Consultant Specialist Veterinary Pathologist, Independent consultant, South Africa

## Abstract

There are few publications on the clinical haematology and biochemistry of African penguins (*Spheniscus demersus*) and these are based on captive populations. Baseline haematology and serum biochemistry parameters were analysed from 108 blood samples from wild, adult African penguins. Samples were collected from the breeding range of the African penguin in South Africa and the results were compared between breeding region and sex. The haematological parameters that were measured were: haematocrit, haemoglobin, red cell count and white cell count. The biochemical parameters that were measured were: sodium, potassium, chloride, calcium, inorganic phosphate, creatinine, cholesterol, serum glucose, uric acid, bile acid, total serum protein, albumin, aspartate transaminase and creatine kinase. All samples were serologically negative for selected avian diseases and no blood parasites were detected. No haemolysis was present in any of the analysed samples. Male African penguins were larger and heavier than females, with higher haematocrit, haemoglobin and red cell count values, but lower calcium and phosphate values. African penguins in the Eastern Cape were heavier than those in the Western Cape, with lower white cell count and globulin values and a higher albumin/globulin ratio, possibly indicating that birds are in a poorer condition in the Western Cape. Results were also compared between multiple penguin species and with African penguins in captivity. These values for healthy, wild, adult penguins can be used for future health and disease assessments.

## Introduction

The African penguin, *Spheniscus demersus*, is listed as an endangered species (International Union for Conservation of Nature [IUCN] 2013), with a > 60% decline in the population between 2001 and 2009. This decrease is primarily attributable to changes in the abundance and availability of prey (Crawford *et al*. 2006; Crawford *et al*. 2011; Sherley *et al*. 2013). Conservation efforts can be aided by the availability of comprehensive health information, which provides baseline data for populations (Heard *et al*. 2013; Karesh & Cook 1995). These baseline clinical data are also critical for monitoring disease as a threat to a species (Heard *et al*. 2013; Karesh & Cook 1995); however, comprehensive health assessments of free-ranging avian species have rarely been reported (Smith *et al*. 2008).

Few publications have reported the clinical haematology and biochemistry of the African penguin, and these are based on captive populations (Cray, Stremme & Arheart 2010; Graczyk *et al*. 1994; Hawkey & Samour 1988; Mazzaro *et al*. 2004; Mazzaro *et al*. 2013; Stoskopf, Yarborough & Beall 1980). In addition, many zoos and aquaria keep in-house reference ranges for clinical haematology and biochemistry, and the International Species Information System (ISIS) maintains a database of medical information on captive wildlife (ISIS 2014). As part of a comprehensive health survey for the African penguin, it was attempted to develop baseline values in clinical haematology and biochemistry for healthy, wild, adult penguins for future health and disease assessments.

## Materials and methods

Between 2010 and 2013, African penguins were sampled as part of a health survey. Healthy, adult penguins were sampled during the breeding season from the Western Cape colonies (Dassen Island, Robben Island, Boulders, Stony Point [Betty's Bay] and Dyer Island) and the Eastern Cape colonies (St. Croix Island and Bird Island). All samples were collected in the winter months (from June to August), except for those from Dassen Island, which were collected in the summer month of December. The birds that were selected were either resting in the colony or sitting on nests with medium-to-large chicks. All birds were bled, measured (head and bill) and weighed. Nest contents were noted and all birds were released back on or close to their nests. Handling time was approximately 5–10 min per bird.

Blood (10 mL – 20 mL) was collected by venipuncture of the jugular vein using a 21-guage needle and immediately transferred into ethylene diamine tetra-acetic acid (EDTA) and serum clot activator tubes (Vacuette^®^ Greiner bio-one, Austria). There was a delay of 4–8 h from collection to processing the samples. In the laboratory, serum clot activator tubes were centrifuged and serum was transferred into separate Eppendorf tubes. Both the EDTA and the serum samples were stored at approximately 4 °C until analysed.

Haematology and biochemistry analyses were all performed by the IDEXX laboratory (Pty) Ltd in Cape Town (except for bile acids, which were sent to the Johannesburg branch). For the Western Cape, samples reached the IDEXX laboratory approximately 24 h after collection and 12 h after serum separation. Samples from Bird Island and St. Croix Island were delayed due to transport from Port Elizabeth to Cape Town. Bird Island samples were ready for processing at the IDEXX laboratory 72 h after collection and 60 h after separation. St. Croix samples were ready for processing 48 h after collection and 36 h after separation. Sex was determined by DNA analysis from blood samples sent to Molecular Diagnostic Services (Pty) Ltd, Durban, South Africa. Serological testing was conducted at the Provincial Veterinary Laboratory in Stellenbosch. Blood smears were made from the EDTA samples in the laboratory at Southern African Foundation for the Conservation of Coastal Birds (SANCCOB, Cape Town, South Africa), air-dried, fixed in methanol for 3 min and stained with modified Wright-Giemsa stain (Kyro-Quick™ stain set, Kyron Laboratories [Pty] Ltd, Benrose, South Africa). All slides were examined for blood parasites at SANCCOB for 10 min, using a 50 X oil immersion lens.

The haematocrit was determined using a Hawksley micro-haematocrit reader (Hawksley, London, England). A Neubauer haemocytometer (Hawksley, London, England) with Turek's fluid was used to determine the total white cell, red cell and thrombocyte counts. The haemoglobin was measured using a Cell-Dyn automated haematology analyser (Abbott Diagnostics, Illinois, USA). Mean corpuscular volume (MCV) and mean corpuscular haemoglobin concentration (MCHC) were calculated from the haematocrit, red cell count and haemoglobin values.

A dry chemistry analyser (Vitros^®^ 350, Ortho Clinical Diagnostics, Raritan, USA) was used for most of the biochemistry values. The colorimetric method was used to analyse calcium, inorganic phosphate, total serum protein, albumin, cholesterol, glucose and uric acid values. The potentiometric method was used to analyse sodium, potassium and chloride values. The two-point rate method was used to analyse creatinine values and the multi-point rate method was used to analyse creatine kinase (CK) and aspartate transaminase (AST) values. The globulin value was calculated from the total serum protein and albumin values. A wet chemistry analyser (ABX Pentra 400, Horiba, Ltd., Kyoto, Japan) was used to analyse serum bile acids with enzymatic colorimetric methodology.

All data were evaluated for normality of distribution, prior to subsequent analysis, using a Shapiro-Wilk test. Mean, standard deviation (s.d.) and range were calculated for each variable in the pooled dataset, in addition to sex and region stratified analysis. Sex was compared between sampling regions using a Pearson Chi-squared test. Differences between sex and region were evaluated using *t*-test, and Mann-Whitney tests were used when normality was rejected. To eliminate potential seasonal variation in haematology and chemistry values, only animals sampled during the winter months were included in the comparison between sampling regions. Statistical significance was set at ≤ 0.05 and all analysis was conducted using SPSS Statistics 20 (IBM Corp., Armonk, NY).

## Results

Between 2010 and 2013, a total of 376 blood samples from African penguins were collected. Only samples from clinically healthy, adult penguins were used in the development of baseline values discussed below. Samples with a positive serological result and/or presence of blood parasites, as well as haemolysed blood samples, were excluded, leaving 108 samples for analysis. The mean haematology and biochemistry values for wild, healthy, adult African penguins taken during the breeding season from the South African colonies are listed in Table 1.

**TABLE 1:** Haematology and blood chemistry values for wild, healthy, adult African penguins sampled during the breeding season in South African colonies.

Variable	Unit	Mean	s.d.	Min	Max	*n*
Head length	mm	121.1	3.89	112.7	131.7	108
Weight	kg	2.86	0.37	2.04	4.16	108
Haematocrit	%	46	5.70	15	55	107
Haemoglobin	g/dL	18.4	2.40	5.5	23.0	103
Red cell count	X 10^12^/L	1.82	0.26	0.64	2.28	103
Mean corpuscular volume	fL	251	35.60	148	534	103
Mean corpuscular haemoglobin concentration	g/dL	40	3.50	31	58	103
White cell count	X 10^9^/L	17.71	8.43	3.50	60.00	103
Sodium	mmol/L	154	6.00	140	168	105
Potassium	mmol/L	5.09	2.52	1.60	19.22	105
Chloride	mmol/L	121	6.10	104	136	104
Calcium	mmol/L	2.77	0.82	1.96	5.91	105
Inorganic Phosphate	mmol/L	1.53	0.62	0.60	3.80	105
Creatinine	µmol/L	24.1	11.89	4.0	54.0	105
Cholesterol	mmol/L	5.36	1.36	1.80	8.80	105
Glucose	mmol/L	11.78	2.15	8.20	19.50	105
Uric Acid	µmol/L	394	221.00	99	1363	104
Bile Acid	µmol/L	9.53	16.75	0.08	78.27	87
Total serum protein	g/L	59.0	9.61	34.0	83.0	105
Albumin	g/L	19.3	3.97	11.0	33.0	105
Globulin	g/L	39.8	6.28	23.0	57.0	105
Albumin/Globulin	-	0.48	0.05	0.37	0.72	105
Aspartate transaminase	U/L	218	90.00	90	627	104
Creatine kinase	U/L	419	272.00	77	1554	105

Standard International (SI) units used.

s.d., standard deviation; Min, Minimum; Max, Maximum.

There were 107 samples that were sexed, with 43 males and 64 females (one sample was not tested). The proportion of males and females between the Eastern (*n* = 48) and Western (*n* = 59) Cape was not significantly different (*p* = 0.36) and were therefore not adjusted when comparing the haematology and biochemistry values across the sampling region.

The total mean body weight was 2.86 kg ± 0.37 kg, with males being significantly heavier than females (Table 1 and Table 2). Measures of head length were also significantly higher among males compared with females (Table 2). The haematocrit, haemoglobin and red cell count values were significantly higher among males compared with females (Table 2). In contrast, females had significantly higher calcium and inorganic phosphate values compared with males (Table 2).

**TABLE 2:** Mean and standard deviations for haematology and blood chemistry values that were significantly different between sexes of African penguins, using an independent sample *t*-test.

Variable	Unit	Male†		Female‡		*p*-value
		Mean	s.d.	Mean	s.d.	
Head length	mm	124.50	3.06	118.90	2.52	< 0.01
Weight	kg	3.08	0.34	2.72	0.31	< 0.01
Haematocrit	%	46.80	5.92	44.10	5.96	0.02
Haemoglobin	g/dL	19.10	2.48	17.80	2.20	0.01
Red cell count	X10^12^/L	1.94	0.25	1.73	0.24	< 0.01
Calcium	mmol/L	2.49	0.22	2.95	1.00	0.01
Inorganic Phosphate	mmol/L	1.33	0.44	1.66	0.69	< 0.01

Standard International (SI) units used.

s.d., standard deviation.

†, *n* = 43; ‡, *n* = 64.

When compared by region of sampling, there were multiple significant differences (Table 3). Penguins in the Eastern Cape weighed more than those in the Western Cape, although the head length was not significantly different (Table 3). Of the haematology and biochemistry values, the white blood cell count, cholesterol and globulin values were higher among birds sampled from the Western Cape; the MCHC, sodium, chloride, phosphorus and albumin/globulin ratio were higher among birds sampled in the Eastern Cape (Table 3).

**TABLE 3:** Mean and standard deviations for all haematology and blood chemistry values that were significantly different by region for African penguins sampled, using an independent sample *t*-test.

Variable	Unit	Western Cape†		Eastern Cape‡		*p*-value
		Mean	s.d.	Mean	s.d.	
Weight	kg	2.80	0.37	2.94	0.36	0.05
Haematocrit	%	46.00	6.70	44.00	4.80	0.02
Mean corpuscular haemoglobin concentration	g/dL	39.00	3.10	42.00	3.50	< 0.01
White cell count	X 10^9^/L	19.98	9.67	14.88	5.47	< 0.01
Sodium	mmol/L	153.00	5.90	156.00	5.90	0.02
Chloride	mmol/L	118.00	6.40	124.00	4.30	< 0.01
Inorganic Phosphate	mmol/L	1.42	0.53	1.66	0.70	0.04
Cholesterol	mmol/L	6.10	1.13	4.44	1.03	< 0.01
Bile Acid	µmol/L	15.35	19.94	1.30	1.29	< 0.01
Globulin	g/L	41.60	6.20	37.60	5.70	< 0.01
Albumin/Globulin	-	0.47	0.05	0.50	0.05	0.02

Standard International (SI) units used.

s.d., standard deviation.

†, *n* = 42; ‡, *n* = 49.

The haematology and biochemistry values were compared with those already published on African penguins (Table 4 and Table 5).

**TABLE 4:** Mean haematology values for the present study and previously published African penguin studies.

Variable	The present study	Mazzaro *et al*. (2013)	Teare (2013)	Graczyk *et al*. (1994)	Hawkey & Samour (1988)	Stoskopf *et al*. (1980)
Sample size	108	21	1048–2884	143	12–42	94
Individuals	Healthy adults (breeding season)	15 adults (outside of molting season)	423–788 healthy adults and juveniles	11 juveniles (non-parasitemic)	Healthy adults	41 adults and juveniles
Area	South African breeding colonies	Mystic Aquarium	37 captive institutions	Baltimore Zoo	England and Scotland zoos	Baltimore Zoo
Haematocrit	46	46	45	43	44	46
Haemoglobin	18.4	-	12.5	14.0	16.8	15.4
Red cell count	1.82	1.70	1.83	2.10	1.74	1.57
Mean corpuscular volume	251	-	238	210	254	-
Mean corpuscular haemoglobin concentration	40	-	29	34	38	33
White cell count	17.7	21.1	15.3	16.0	9.3	11.6

Standard International (SI) units used.

Note: Please see the full reference list of the article, Parsons, N.J., Schaefer, A.M., Van der Spuy, S.D. & Gous, T.A., 2015, ‘Establishment of baseline haematology and biochemistry parameters in wild adult African penguins (*Spheniscus demersus*)’, *Journal of the South African Veterinary Association* 86(1), Art. #1198, 8 pages. http://dx.doi.org/10.4102/jsava.v86i1.1198, for more information.

**TABLE 5:** Mean biochemistry values for the present study and previously published African penguin studies.

Variable	The present study	Mazzaro *et al*. (2013)	Teare (2013)	Cray *et al*. (2010)	Mazzaro *et al*. (2004)	Graczyk, Cranfield & Bicknese (1995)
Sample size	108 (serum)	21 (plasma)	1048–2884	20 (plasma)	65 (plasma)	17 (serum)
Individuals	Healthy adults (breeding season)	15 adults (outside of molting season)	423–788 healthy adults and juveniles	Healthy adults (pre-feeding)	Adults	9 uninfected juveniles
Area	South African					
breeding colonies	Mystic Aquarium	37 captive institutions	Adventure Aquarium	6 captive USA institutions	Baltimore Zoo	
Sodium	154	155	155	-	153–156	-
Potassium	5.1	5.2	4.5	-	3.1–4.6	-
Chloride	121	116	116	-	110–115	-
Calcium	2.77	3.00	2.62	-	-	-
Inorganic Phosphate	1.53	0.97	1.17	-	-	0.84
Creatinine	24	12	40	-	-	36
Cholesterol	5.36	7.27	7.07	-	-	-
Glucose	11.78	12.49	12.09	10.87	-	-
Uric Acid	394	-	516	488	-	360
Bile Acid	9.53	-	-	7.1	-	-
Total serum protein	59	-	53	63	-	-
Albumin	19	19	18	-	-	-
Globulin	40	31	32	-	-	-
Albumin/Globulin	0.48	0.62	0.56	-	-	-
Aspartate transaminase	218	173	164	301	-	-
Creatine kinase	419	237	362	598	-	-

Standard International (SI) units are used with conversion table used to convert from conventional units (Ritchie, Harrison & Harrison 1994).Note: Please see the full reference list of the article, Parsons, N.J., Schaefer, A.M., Van der Spuy, S.D. & Gous, T.A., 2015, ‘Establishment of baseline haematology and biochemistry parameters in wild adult African penguins (*Spheniscus demersus*)’, *Journal of the South African Veterinary Association* 86(1), Art. #1198, 8 pages. http://dx.doi.org/10.4102/jsava.v86i1.1198, for more information.

## Ethical considerations

Research permits to conduct this work were obtained by the Department of Environmental Affairs (RES2012/61 EXT, RES2011/19, RES2010/58), CapeNature (AAA007-00047-0056, AAA004-0508-0035) and South African National Parks (PARSN1027).

## Discussion

The presence of more female (60%) than male (40%) birds in the results is probably related to sample condition rather than an actual skewed sex ratio. This is based on the fact that only 107 samples could be used for analysis; however, a total of 165 adult penguins were sampled in the various colonies, with 52% recorded as female and 48% recorded as male (N.J.P. unpublished data). The birds were selected randomly during the breeding season, either sitting with medium-to-large chicks, or resting in the colony. The African penguin parents share incubation and chick-rearing responsibilities (Rand 1960); therefore, the ratio would be expected to be equal.

The mean weight of an adult African penguin (during the breeding season) was similar to those previously recorded (Cooper 1978; Rand 1960); the male being slightly larger than the female (Cooper 1972; Pichegru *et al*. 2013; Rand 1960). The birds in the Eastern Cape were significantly heavier than those in the Western Cape, but not with significantly different head lengths (indicating that birds are structurally the same size in the different regions) and, therefore, in better body condition. Labocha and Hayes (2012) showed that body weight alone is as good an indicator of fat content as any other morphometric condition index, especially if birds are similar in structural size, gut content mass, hydration and organ and muscle mass. In the present study, breeding birds were all bled during the morning and were therefore likely to have similar hydration and gut content mass. It has been documented that foraging adults return in the afternoon to feed their chicks (Frost *et al*. 1976).

When sampling birds, the sample of choice is plasma, due to the larger volume of plasma that can be harvested compared with serum, and the risk of the serum clotting after separation (Campbell 2004; Harr 2006; Lumeij 2008). Samples should be centrifuged immediately after collection in order to separate the plasma from the cells in order to limit changes that occur from haemolysis and cellular metabolism (Harr 2006; Hochleithner 1994; Hrubec *et al*. 2002; Lumeij 2008). There are many practical difficulties when sampling birds in the wild, resulting in datasets that are incomparable due to the different procedures used (Low *et al*. 2006). In the present study, serum was used for analysis due to the expected delay in centrifugation, with the rationale that the clot activator containers would effectively separate the serum from the cells before centrifugation. In this way, it was hoped that the results for wild African penguins would have minimal artificial changes. In health surveys conducted in other penguin species, serum was also used for biochemistry analysis of the Galápagos penguin, *Spheniscus mendiculus*, (Travis *et al*. 2006) and plasma was used for the Humboldt penguin, *Spheniscus humboldti*, (Smith *et al*. 2008) and Rockhopper penguin, *Eudyptes chrysocomes*, (Karesh *et al*. 1999). The findings in the present study were not always consistent with those of Hrubec *et al*. (2002), when comparing serum and plasma, indicating that analysing the results may be dependent on other, often unknown, variables.

In general, the red cell values were comparable to those previously published in African (see references in Table 4) and other penguin species (Hawkey *et al*. 1989; Hawkey & Samour 1988; Karesh *et al*. 1999; Moreno *et al*. 2002; Sergent *et al*. 2004; Smith *et al*. 2008; Travis *et al*. 2006; Villouta *et al*. 1997). The haematocrit is a stable value between studies and bird species (Hawkey & Samour 1988). Likewise, across the spheniscid species, the haematocrit has varied between 41%, recorded for captive adult Humboldt penguins recently removed from the wild (Villouta *et al*. 1997), and 48%, recorded for captive adult Humboldt penguins (Hawkey & Samour 1988). There is no explanation for the significantly lower haematocrit value (44%) in the Eastern Cape birds compared with the Western Cape birds (46%), but both values are well within the normal clinical range, as reported by Campbell (1994).

The mean haemoglobin value of 18.4 g/dL found in the present study was the highest recorded (together with the MCHC) across penguin species (Graczyk *et al*. 1994; Hawkey *et al*. 1989; Hawkey & Samour 1988; Sergent *et al*. 2004; Stoskopf *et al*. 1980; Villouta *et al*. 1997), but no explanation was found for this. It is possible that the delay between collection and analysis, as well as the laboratory methods used, influenced the result. Sergent *et al*. (2004) supported findings that deeper-diving penguin species had higher haemoglobin and haematocrit values compared with shallower-diving penguin species, due to increased oxygen-carrying capacity; however, African penguins are shallow-diving penguins (Ryan *et al*. 2007) and do not conform to this finding. Due to the difference in red blood cell size, the red cell count varies among animal species (higher counts with smaller cells so that the haematocrit is maintained within narrow limits) (Hawkey & Samour 1988). The size of the red cell of the African penguin (mean MCV 251 fL) is relatively large among the penguin species (Hawkey & Samour 1988; Hawkey *et al*. 1989). Teare (2013) and Graczyk *et al*. (1994) reported lower MCV values for the African penguin, but this can be explained by the fact that their studies also included juvenile birds, and cells are smaller in immature birds (Hawkey & Samour 1988).

There are few studies that report sexual differences in red cell values of different bird species, and none in African penguins. In the present study, the haematocrit, haemoglobin and red cell counts were significantly higher in males compared with females. A higher haematocrit in males has also been reported as a non-significant finding in Magellanic penguins (Moreno *et al*. 2002) and as a significant finding in Galápagos penguins (Travis *et al*. 2006). Both values in the present study (male 47%, female 44%) are well within normal clinical values (Campbell 1994).

In the present study, the mean white cell count is at the top end of the range of those recorded from captive African penguins, with this value showing large variation within this species. A high white cell count could indicate a stress response, inflammatory response, or exposure to disease or contaminants (Campbell 1994; Davis *et al*. 2008; Hawkey & Samour 1988; Newman *et al*. 2007). The white cell count did not show any significant difference between the sexes, but did show a significant difference regionally. The white cell count was significantly higher in the Western Cape birds and this could indicate higher stress levels or exposure to disease, compared with the Eastern Cape birds; this is supported by the poorer condition of these birds. Leucocyte profiles can provide reliable assessments of stress in all vertebrates (Davis *et al*. 2008) and further study (as well as differential cell counts) in the African penguin is needed to elucidate this point.

The biochemistry values were compared with those of other published African penguin studies (Table 5) as well as to other penguin species (Aguilera *et al*. 1993; Ghebremeskel *et al*. 1989; Karesh *et al*. 1999; Smith *et al*. 2008; Travis *et al*. 2006; Villouta *et al*. 1997; Wallace *et al*. 1995). The electrolytes (sodium, potassium and chloride) are maintained within narrow limits (Carlson & Bruss 2008). This is due to the fluid and electrolyte balance needed within the body to maintain membrane and electrical stability, and enzymatic-mediated metabolic reactions that sustain normal physiological and biochemical processes (Carlson & Bruss 2008; Harr 2006). There was greater variation in these values between different species compared with between the African penguin studies, although the potassium and chloride values were slightly higher in the present study. The potassium value is higher in serum compared with plasma (Hochleithner 1994) and is affected by time and temperature (Abou-Madi & Jacobson 2003; Campbell 2004; Carlson & Bruss 2008; Mazzaro *et al*. 2004; Rehak & Chlang 1988). Hrubec *et al*. (2002) found lower potassium and chloride values in serum in chickens, compared with plasma, which contradicts the findings of the present study. There is no explanation for the high chloride value in apparently normal, healthy penguins. There is generally little variation in chloride values (Hochleithner 1994). No explanation was found for the regional differences in sodium and chloride values in the present study. Mazzaro *et al*. (2004) did not find significant differences in sodium values between captive birds with or without salt supplementation, or between wild and captive African penguins; however, the potassium and chloride values differed due to unknown or uncontrolled variables. Wallace *et al*. (1995) found an unexplained seasonal difference in chloride values.

Similarly, the calcium and inorganic phosphate values showed little variation within and between species. Reproductively active females have higher calcium values compared with males (Campbell 2004; Hochleithner 1994; Newman *et al*. 1997), as seen in the present study. Smith *et al*. (2008) also reported higher calcium and phosphate values in females, compared with male, wild Humboldt penguins. The calcium and phosphorus values both increase during egg formation with a calcium:phosphorus ratio of above one in a healthy penguin (Harr 2006). Phosphorus values have poor diagnostic value due to inconsistent changes caused by disease conditions (Hochleithner 1994). The creatinine value showed large variation within and between species and has poor diagnostic value in birds (Campbell 2004; Hochleithner 1994). The cholesterol value in the present study was lower than that in the captive African penguin studies, but it was similar to those in other wild penguin studies; this may be explained by the higher fat diet and sedentary life in captivity. Cholesterol values vary with a bird's diet, being higher in carnivorous birds compared with fruit-eating or grain-eating birds (Hochleithner 1994), and high in seabirds, based on the high fat content of fish consumed by these species (Newman *et al*. 1997). The glucose values ranged from 10.87 mmol/L (Cray *et al*. 2010) to 15.9 mmol/L (Aguilera *et al*. 1993) in the African penguin, and this variation between studies was most likely due to sample handling and time between sampling and processing (Hochleithner 1994). In previous penguin studies, the glucose value showed no significant difference due to feeding (Cray *et al*. 2010), post capture (Villouta *et al*. 1997) or during the moult fast (Cherel & Le Maho 1988). Glucose is maintained at adequate levels in plasma, and pathological changes are rarely detected (Hochleithner 1994).

Bile acids are used as a specific and sensitive indicator of liver function in birds (Campbell 2004; Harr 2006; Hochleithner 1994; Lumeij 2008). The bile acid value showed large variation in the present study and could only be compared with one other study in any penguin species (Cray *et al*. 2010). Unfortunately, radio-immunoassay (RIA) methods, as used to determine bile acid levels by Cray *et al*. (2010), cannot be compared with those obtained by enzymatic methods, as used in the present study, due to lower values generated by RIA (Harr 2006; Hochleithner 1994). Cray *et al*. (2010) reported significant changes in both uric acid and bile acid values due to feeding, recommending the need to perform blood assessments at a specified fasted period to minimise artifacts (also noted by Kolmstetter & Ramsay [2000] for uric acid); however, this is not always possible in wild animals. These values, therefore, have limited value in wild birds, unless a fasted period is known to have occurred. There was a large variation in uric acid values within and between penguin species. Uric acid is widely used in birds, mainly as an indicator of renal disease as well as starvation (Hochleithner 1994), and is also high in seabirds, due to a fish diet and living in a marine environment (Newman *et al*. 1997). It is not a sensitive or specific indicator of renal disease and is only elevated once the renal function is severely compromised (Campbell 2004; Lumeij 2008).

Total protein is higher when testing plasma compared with serum, due to the presence of fibrinogen (Hochleithner 1994; Lumeij 2008); however, the values are similar to other studies where plasma was used. In general, the total protein ranged between 50 g/L and 60 g/L in the African and other penguin species, with albumin lower than the total globulin values. Karesh *et al*. (1999) reported the lowest total protein value of 35 g/L in Rockhopper penguins. Total protein is often used as a health indicator in birds, but levels are influenced by many physiological factors (age, breeding stage, diet, temperature) as well as pathological changes (chronic disease, blood loss, malnutrition, chronic infection, dehydration) (Harr 2006; Hochleithner 1994). Protein values are used in clinical practice to evaluate the nature, severity and progress of disease, but not to provide a specific diagnosis (Lumeij 2008). Lumeij (2008) and Harr (2006) questioned the validity of using dye-binding techniques instead of electrophoresis in determining albumin and globulin levels in birds, as well as the need to validate the individual species tested against the standard used in the laboratory. These values should, therefore, be used with caution when comparing with other species and other studies. The albumin/globulin ratio showed large variation within and between species, but showed little variation in the present study, at approximately 0.5. The albumin/globulin ratio is of greater clinical importance than the total protein value, as the inflammatory process causes a rise in globulins, often together with a decrease in albumin values (Hochleithner 1994; Lumeij 2008). There was no significant difference in the total protein values when comparing the Western Cape and Eastern Cape birds; however, the globulin values were higher in the Western Cape birds, with a lower albumin/globulin ratio compared with the Eastern Cape birds.

The two enzymes tested in the present study were AST and CK. Aspartate transaminase is present in many tissues, but generally reflects activity in the muscle and liver, whilst CK is specific for muscle activity (Campbell 2004; Harr 2006; Hochleithner 1994; Lumeij 2008). In the present study, there was large variation in both of these results, limiting their value, and this was also seen when comparing within and between other penguin species. Enzyme levels serve as a rough guide, due to different removal rates from the blood, for example, CK has a shorter half-life than AST and, therefore, biochemical values need to be evaluated together (Lumeij 2008).

The Western Cape penguins weighed less, with higher white cell counts, higher globulin values and a lower albumin/globulin ratio when compared with the Eastern Cape penguins. This could indicate that penguins in the Western Cape are in poorer condition, with possible evidence of inflammation, higher stress levels or exposure to disease. This conclusion is supported by research into population numbers, chick condition and foraging data. African penguin numbers have been declining since 2001, with a 50% decline in the Eastern Cape and an 80% decline in the Western Cape (Crawford *et al*. 2011; Pichegru *et al*. 2014). Although the numbers are still decreasing in the Eastern Cape, the rate of decrease in numbers of penguins breeding is slower compared with the Western Cape. Algoa Bay hosts more than half of the South African penguin population, with St. Croix Island currently the largest global African penguin colony (Pichegru *et al*. 2014). In 2008 and 2009, African penguin chick growth was better in the Eastern Cape colonies compared with those on Robben, Dassen and Dyer islands (Sherley 2010). Chick condition was better in the Eastern Cape colonies in 2008 compared with the Western Cape colonies; however, the results in 2009 were related to individual colonies and not breeding region (L.J. Waller unpublished data). Adult condition and environmental conditions determine chick provisioning rates (Ballard *et al*. 2010) and, therefore, chick condition and growth can be viewed as a function of adult condition. Penguins breeding on Dassen Island (Western Cape) had longer foraging-trip lengths and took on average 6 days longer to reach the necessary condition before moult, compared with penguins on Bird Island (Eastern Cape) (Harding 2013). The results in the present study were based on once-off sampling at each colony, and repeated sampling over several years would help to elucidate whether differences could be assigned to particular years, environmental or fishing conditions particular to each colony and breeding region.

These results are valid only for African penguins, with the methodology and laboratories used in the present study. There is variation between different laboratories, different methodologies and sampling handling that make determining reference intervals unreliable (Harr 2006). Normal values and reference ranges should be used with caution, due to different sample sizes, test methodologies, statistical analyses used, as well as the evaluation of ‘healthy’ and inherent variability (Lumsden 1998).

This is the first report of baseline clinical haematology and biochemistry values of healthy, wild, adult African penguins in South Africa. The results are important data to aid in the study of health and disease of this population. Further research into the pathophysiological effects of avian diseases on this population, using comprehensive health assessment data, is needed to fully understand the threats facing this endangered species.
